# Evaluation of serum neopterin levels in severe COVID-19 patients: An observational study

**DOI:** 10.1097/MD.0000000000038996

**Published:** 2024-07-26

**Authors:** Sinem Gürcü, Zeynep Irmak Kaya, Ali Uncu, Göknur Yorulmaz, Sinem Ilgin

**Affiliations:** a Department of Pharmacy, Eskişehir City Hospital, Eskişehir, Turkey; b Department of Internal Medicine, University of Health Sciences, Eskişehir Health Application and Research Center, Eskişehir, Turkey; c Department of Medical Biochemistry, Department of Basic Medical Sciences, University of Health Sciences Eskişehir Health Application and Research Center, Eskişehir, Turkey; d Department of Endocrinology, Faculty of Medicine, Eskisehir Osmangazi University, Eskişehir, Turkey; e Department of Pharmaceutical Toxicology, Faculty of Pharmacy, Anadolu University, Eskişehir, Turkey.

**Keywords:** COVID-19, inflammation, inflammatory markers, neopterin, pteridines

## Abstract

In patients with coronavirus disease (COVID-19), a massive inflammatory response is a significant cause of morbidity and mortality. Inflammatory markers are prognostic indicators of disease severity and the ultimate clinical outcome. Several studies have demonstrated a correlation between serum levels of neopterin, which can be an immune system marker, disease severity, and poor outcomes in COVID-19 patients. Our study aimed to determine the diagnostic significance of neopterin in conjunction with routinely measured inflammatory markers in patients with severe COVID-19. Serum neopterin, C-reactive protein (CRP), albumin levels, and complete blood count were determined in 39 patients with severe COVID-19 and 30 healthy individuals. Demographic characteristics, serum neopterin levels, and other laboratory data were compared between patients and healthy volunteers and statistically analyzed. High neopterin levels were observed in patients with severe COVID-19 compared to healthy volunteers. Furthermore, albumin levels were decreased, while CRP levels were increased in patients, statistically significantly. Also, positive correlations were shown between serum neopterin levels and serum CRP levels, while negative correlations were shown between serum neopterin levels and serum albumin levels. Systemic inflammation markers, CRP/albumin ratio, neutrophil/lymphocyte ratio, and platelet/lymphocyte ratio were significantly higher, while lymphocyte/monocyte ratio was also significantly lower in patients with severe COVID-19 than in healthy volunteers. However, serum neopterin levels were not linked to the CRP/albumin ratio, the neutrophil/lymphocyte ratio, or the platelet/lymphocyte ratio. On the other hand, they were linked negatively to the lymphocyte/monocyte ratio. Our findings highlight the association between high neopterin levels and patients with severe COVID-19. Neopterin is correlated with traditional inflammatory biomarkers and may indicate general immune and inflammatory activation in patients with severe COVID-19.

## 1. Introduction

COVID-19, announced as a global epidemic in 2020 by the World Health Organization (WHO), has affected an estimated 769 million individuals, resulting in the loss of approximately 7 million lives to date.^[1]^ A new enveloped RNA beta-coronavirus, severe acute respiratory syndrome coronavirus 2 (SARS-CoV-2), has been defined as the causative pathogen of the disease. The clinical course of COVID-19 is a broad spectrum ranging from asymptomatic disease to death.^[2,3]^ Age, male gender, and preexisting comorbidities increase the risk of disease severity or mortality.^[2,4–6]^ It is known that the hyperinflammatory syndrome is a prominent contributor to disease severity and mortality in COVID-19 patients.^[3,6–8]^ It is thought that SARS-CoV-2 elicits a localized immune response by aggregating monocytes and macrophages at the site of infection, thereby inducing cellular immunological activation.^[9]^ The pro-inflammatory cytokines interferon-gamma (IFN-γ) and interleukin-6 (IL-6) are crucial in coordinating inflammatory cascades and have a significant elevation in COVID-19 patients.^[3,6–8]^ Recognized inflammatory biomarkers play an essential role in managing patients with COVID-19, including diagnosis, prognosis, and assessment of treatment response.^[10]^ Inflammatory markers, including complete blood count, C-reactive protein, albumin, cytokines, and erythrocyte sedimentation rate, have been linked to the severity and mortality of COVID-19.^[10–12]^ However, no specific biomarker for patients to progress to severe or even death has been identified. Therefore, it has been focused on determining accurate biomarkers that may assist in predicting illness progression and developing effective treatment strategies in the latest studies. Neopterin is 1 of the focused biomarkers for predicting the diagnosis and progress of COVID-19 disease. Studies have demonstrated the association between neopterin levels and the severity of the disease and poor clinical course in patients with COVID-19.^[4,5,7,8,13–15]^ Neopterin is a widely recognized biomarker of immunological activation that exhibits increased levels in many inflammatory conditions. Infections induce the production of IFN-γ, which triggers the activation of monocytes and macrophages, leading to the synthesis of neopterin.^[4,16]^ Therefore, increasing neopterin levels in COVID-19 patients is unsurprising.

In this study, we determined the profile of traditional biomarkers of inflammation, including CRP, albumin, complete blood count, and neopterin levels, in patients with COVID-19 admitted to the intensive care unit (ICU) and in healthy volunteers. At this point, few studies showed a correlation between traditional biomarkers of inflammation and neopterin. Also, the present study evaluated the correlations of routine laboratory biomarker levels with neopterin levels in COVID-19 patients.

## 2. Materials and methods

### 2.1. Study design

Power analysis was performed to calculate the sample size, and power analysis was calculated at a 95% confidence and 5% error level. Based on this calculation, approximately 35 patients were included in the study. The study included 39 patients diagnosed with severe COVID-19 who were followed up in the ICU of Eskişehir City Hospital and 30 healthy volunteers.

The study was approved by the Non-Interventional Clinical Research Ethical Committee of Eskişehir Osmangazi University (date: September 28, 2021, #9). This study was conducted in accordance with the Declaration of Helsinki. All information about the study was provided to the relatives of the patients, and written consent was obtained from all first-degree relatives.

### 2.2. Study population

Between November 1 and November 30, 2021, 119 COVID-19 patients were followed up in the Eskişehir City Hospital Intensive Care Unit. The patients were diagnosed according to the WHO criteria and positive reverse transcriptase polymerase chain reaction (RT-PCR) testing of nasopharyngeal and throat aspirate samples. Patients typically had computed tomography images of viral pneumonia, such as ground-glass attenuations. The patients received no COVID-19 vaccination, tocilizumab, or intravenous vitamin C or immunoglobulin treatment. Permission to participate in the study was obtained from patients’ relatives. It was determined from hospital records that the patients received pulse steroid (methylprednisolone IV, 250 mg) treatment for 3 days before they were transferred to the ICU. Patients with serum creatinine levels > 2.0 mg/dL, secondary bacterial infections, cancer, autoimmune diseases, and those under the age of 18 were excluded from the study. The control group comprised healthy volunteers who were not vaccinated or had no disease.

Consequently, the prospective study sample included 39 COVID-19 patients attending the ICU and 30 healthy controls.

### 2.3. Study protocol

To determine neopterin, blood samples were taken from the participants before the first feeding in the morning, centrifuged at 3500 rpm for 10 minutes, and then serum specimens were stored at −80 °C until neopterin measurement. As reported by the manufacturer’s instructions, commercial enzyme-linked immunosorbent assay (ELISA) kits (Human Neopterin Assay Kit, Bioassay Technology Laboratory, China) were used to determine serum neopterin concentrations. Optical density was measured at 450 nm using a microplate reader (Spectra Max M2, UK). The serum neopterin levels were measured at nmol/L. Additionally, CRP and albumin levels were measured in serum samples prepared from the blood. This analysis was performed using an Architect C8200 Integrated System Clinical Chemistry and Immunoassay Analyzer (Abbott Diagnostics, USA) at the Medical Biochemistry Laboratory Diagnosis of the Eskisehir City Hospital.

Complete blood count analysis was performed using Sysmex XN 1000 hematology analyzers (Sysmex Corporation, Kobe, Japan) at the Medical Biochemistry Laboratory Diagnosis of the Eskisehir City Hospital, and neutrophil, lymphocyte, monocyte, leukocyte, and platelet counts were measured.

The CRP/albumin ratio, lymphocyte–monocyte ratio, neutrophil/lymphocyte ratio (NMR), and platelet/lymphocyte ratio (PLR), which are systemic inflammation markers, were calculated by dividing them by each other.^[17]^

### 2.4. Statistics

SPSS 20 version (SPSS Inc., Chicago, IL, USA) was used for statistical analysis. While evaluating the study data, in addition to descriptive statistical methods (Mean, Standard Deviation, Median, Frequency, Ratio, Minimum, Maximum), the Independent Sample T-test was used for the comparison of normally distributed parameters between the 2 groups, and Pearson Chi-Square test was used for the comparison of qualitative data. Correlation analysis was performed using the Number Cruncher Statistical System 2007 (Kaysville, Utah, USA) program. Pearson correlation analysis was performed to determine the relationship between measurements. A *P*-value of <.05 was considered statistically significant.

## 3. Results

Of the patients with severe COVID-19 included in the study (n = 39), 46.2% were female and 53.8% were male. Gender differences across the groups were not significant (*P* = .751). The mean age was 60.79 ± 17.49 years for patients with severe COVID-19 and 33.87 ± 10.82 for healthy volunteers. There was a statistically significant difference between the groups regarding mean age. The participants’ data are presented in Table 1.

**Table 1:** Demographical and laboratory findings of severe COVID-19 patients and healthy volunteers.

		Groups				*P*
		Control		Severe COVID-19		
		n	%	n	%	
Gender	Male	15	50.0	21	53.8	.751^a^
	Female	15	50.0	18	46.2	

		Mean ± Sd	Min–Max (Median)	Mean ± Sd	Min–Max (Median)	
Age		33.87 ± 10.82	18 to 63 (32.5)	60.79 ± 17.49	31 to 90 (64)	.001**^b^
Lymphocyte (×10^3^/μL)		2.56 ± 0.74	1.47 to 3.9 (2.4)	1.07 ± 0.65	0.21 to 2.74 (0.86)	.001**^b^
Monocyte (×10^3^/μL)		0.58 ± 0.2	0.23 to 1.03 (0.53)	0.65 ± 0.35	0.17 to 1.84 (0.56)	.311^b^
Neutrophil (×10^3^/μL)		4.44 ± 1.14	1.98 to 6.27 (4.27)	11.06 ± 4.05	4.15 to 22.84 (10.98)	.001**^b^
Leukocyte (×10^3^/μL)		7.59 ± 1.47	5.34 to 10.82 (7.61)	13.47 ± 3.74	6.3 to 24.38 (12.69)	.001**^b^
Platelet (×10^3^/μL)		247.2 ± 55.04	136 to 354 (248.5)	224.26 ± 70.17	96 to 426 (222)	.145^b^
Albumin (g/L)		46.8 ± 2.11	41 to 51 (47)	29.83 ± 3.91	20.5 to 41.3 (29.25)	.001**^b^
CRP (mg/L)		0.89 ± 2.15	0 to 11 (0.25)	81.13 ± 70.74	1.9 to 271.1 (63.1)	.001**^b^
CRP/albumin ratio		0.02 ± 0.04	0 to 0.22	2.82 ± 2.63	0.05 to 11.46	.001**^b^
Neutrophil/lymphocyte ratio		1.86 ± 0.65	0.68 to 3.33	14.72 ± 10.22	1.79 to 41.19	.001**^b^
Platelet/lymphocyte ratio		101.66 ± 26.79	53.58 to 145.28	284.88 ± 184.38	37.65 to 961.90	.001**^b^
Lymphocyte/monocyte ratio		4.71 ± 1.58	2.41 to 8.14	1.90 ± 1.29	0.29 to 7.08	.001**^b^
Neopterin (nmol/L)		2.26 ± 2.13	0.51 to 12.62 (1.85)	16.57 ± 9.29	1.76 to 32.13 (15.21)	.001**^b^

a Pearson Chi-square.

b Independent Sample *T*-Test.

** *P* < .01.

**Figure 1.**
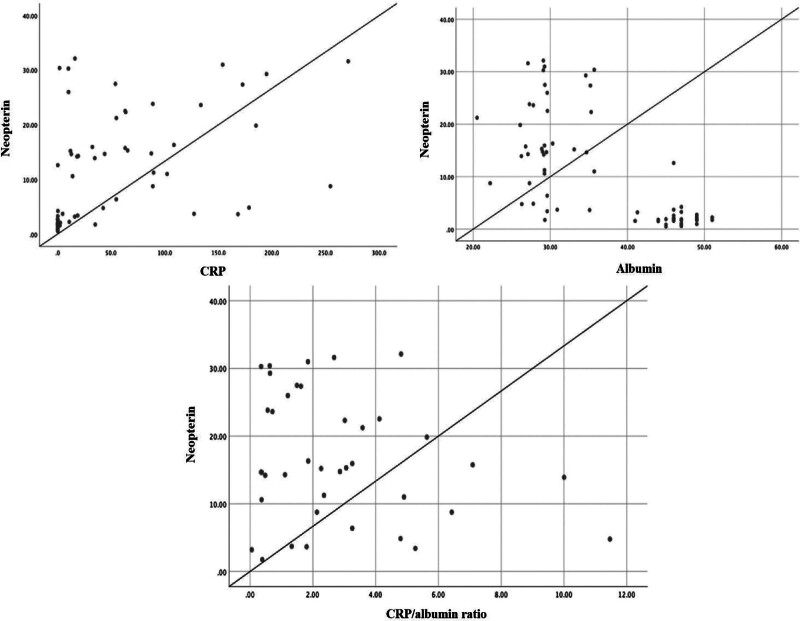
Correlation between serum neopterin levels and CRP, albumin, and CRP/albumin ratio in patients with severe COVID-19.

Patients with severe COVID-19 had a statistically significant increase in neopterin levels compared to those in healthy volunteers (mean value 16.57 ± 9.29, 2.26 nmol/L ± 2.13, respectively) (*P* < .001). Also, according to healthy controls, the patients had higher CRP levels and lower albumin levels (*P* < .001). Additionally, a higher CRP/albumin ratio was observed in the patients than in healthy volunteers (*P* < .001). Also, according to healthy controls, the patients had higher CRP levels and lower albumin levels (*P* < .001). Additionally, a higher CRP/albumin ratio was observed in the patients than in healthy volunteers (*P* < .001). We analyzed correlations between neopterin levels, CRP, albumin levels, and the CRP/albumin ratio in severe COVID-19 patients. We found that a positive correlation was between neopterin and CRP levels (*r* = 0.485, *P* < .001), while a negative correlation was between neopterin and albumin levels in patients with severe COVID-19 (*r* = 0.669, *P* < .001). There was no correlation between neopterin level and CRP/albumin ratio (*r* = 0.222, *P* = .174) (Fig. 1).

**Figure 2.**
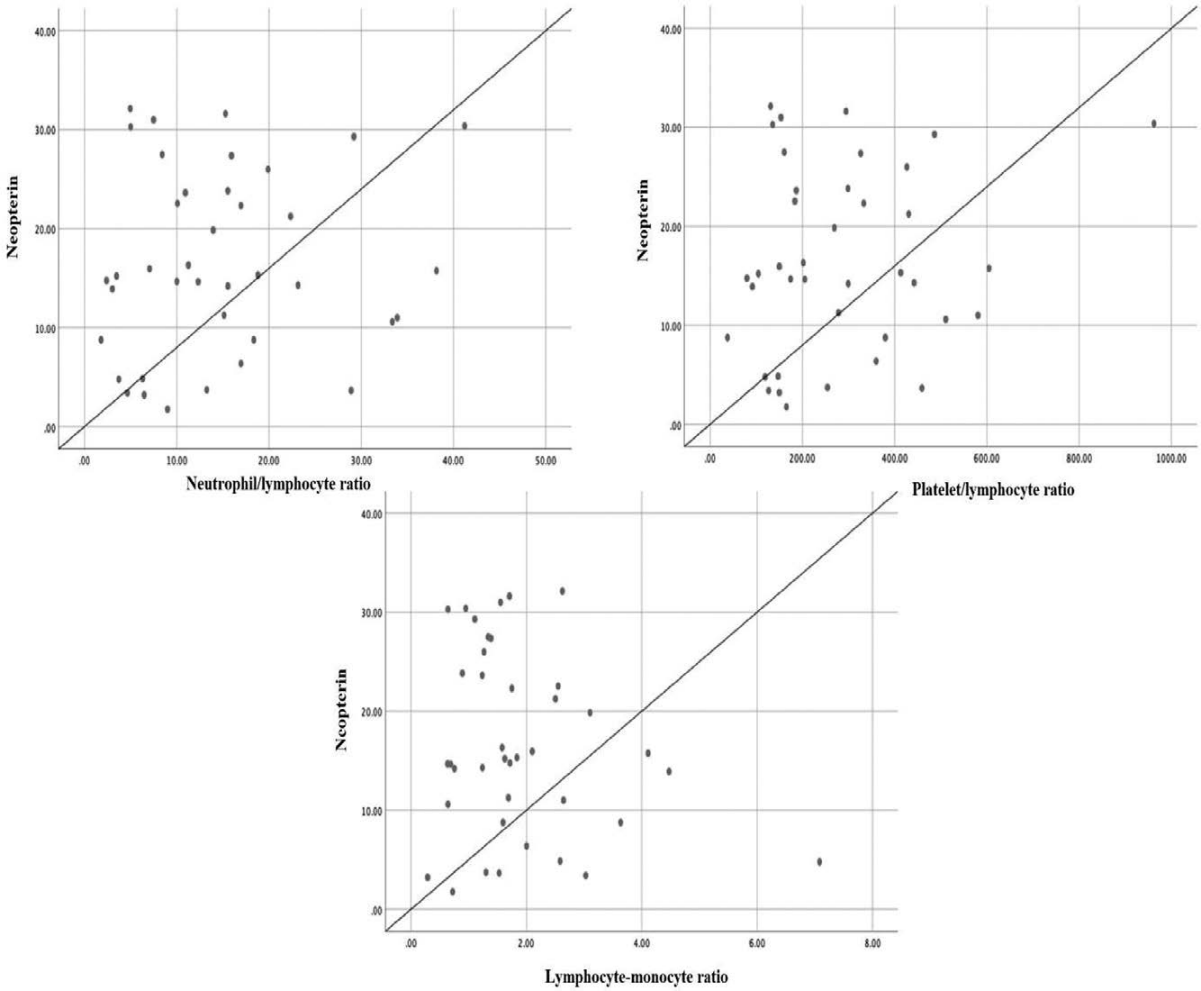
Correlation between serum neopterin levels and neutrophil/lymphocyte ratio, platelet/lymphocyte ratio, and lymphocyte/monocyte ratio in patients with severe COVID-19.

Patients with severe COVID-19 had lower leukocyte, neutrophil, and lymphocyte counts than healthy volunteers (*P* < .001). Also, a higher neutrophil/lymphocyte ratio, a lower platelet/lymphocyte ratio, and a lower lymphocyte/monocyte ratio were observed in the patients compared to healthy volunteers. Neopterin levels did not correlate with neutrophil/lymphocyte ratio, platelet/lymphocyte ratio, or lymphocyte/monocyte ratio (*r* = 0.117, *P* = .477; *r* = 0.176, *P* = .284; *r* = 0.211, *P* = .197, respectively) (Fig. 2).

## 4. Discussion

Neopterin has been proposed as an important indicator of monocyte/macrophage axis activation in a wide range of conditions. Elevated levels of neopterin in biological fluids have been linked to a wide range of disorders that involve the activation of the cellular immune system, including viral and bacterial infections, autoimmune diseases, and malignant tumors.^[4,18–20]^ Recent studies indicate that assessing neopterin levels could contribute to the diagnosis and treatment course of COVID-19. The pathophysiology of COVID-19 is attributed to an exaggerated inflammatory response. The excessive inflammatory response leads to the unregulated proliferation of monocytes and macrophages, which is mediated by IFN-γ and results in organ damage. The release of IFN-γ from T cells leads to an elevation in neopterin levels.^[15]^ Therefore, neopterin may serve as an indicator of the cell-mediated immune system due to its association with IFN-γ. Our findings indicated that patients with severe COVID-19 exhibited elevated neopterin levels, a marker of immune activation, compared to healthy volunteers. Other research has also attracted attention to elevated serum neopterin levels in patients with severe COVID-19.^[2,4,5,7,13,15]^ Furthermore, a more significant elevation of neopterin levels in COVID-19 patients than in healthy individuals was reported to be approximately 8-fold in our study (2.26 ± 2.13 vs 16.57 ± 9.29). On the other hand, our findings indicated that CRP levels were increased in patients with severe COVID-19. Clinical studies also demonstrated that CRP levels are at increased levels in COVID-19 patients and have been correlated with the course and prognosis of the disease.^[21–27]^ CRP is a protein produced in response to inflammation or infection. It is primarily triggered by the action of IL-6 on the gene responsible for producing CRP during the acute phase of the inflammatory/infectious process. CRP plays a crucial role in the body’s first line of innate defenses to identify and remove harmful microorganisms and damaged cells by activating the complement system and phagocytic cells.^[28–30]^ So, CRP is an early marker of infection and inflammation. Also, Bellmann-Weiler et al^[4]^ demonstrated a positive correlation between CRP and neopterin levels and the severity of COVID-19. Similarly, in our study, a positive correlation was observed between elevated neopterin levels and increased CRP levels in patients with severe COVID-19. A positive correlation was also observed between neopterin and CRP levels in different pathology related to immunity and inflammatory status.^[31–33]^ Additionally, in our study, low albumin concentration was also detected, in addition to the systemic inflammatory status indicated by high neopterin and CRP levels. Studies also indicated that hypoalbuminemia was an independent and early predictor of poor prognosis in patients with COVID-19.^[17,25–27,34–36]^ Hypoalbuminemia is a negative acute-phase reactant linked to an inflammatory response and an unfavorable prognosis in viral illnesses. When cytokines and chemokines are released, they make capillaries more permeable. This changes how albumin is distributed between the inside and outside of blood vessels.^[34,35]^ A negative correlation between CRP and albumin levels in COVID-19 patients, a sign of inflammatory status, has been demonstrated.^[17,34,35]^ Similar to the relationship between CRP and albumin levels, a significantly negative correlation was found between neopterin and albumin levels in patients with severe COVID-19 in our study. Also, the CRP/albumin ratio is calculated by dividing the CRP value by the albumin measurement. It is a recognized scoring system that helps assess the extent and intensity of inflammatory diseases. This ratio is regarded as a more informative predictor of inflammation than CRP or albumin alone.^[37,38]^ Studies indicated that higher CRP/albumin ratios in COVID-19 patients were associated with an increased severity or mortality.^[17,39–42]^ Our study noted that the CRP/albumin ratio was significantly higher in patients with severe COVID-19 than healthy controls.

Recently, novel markers derived from complete blood counts linked to the inflammatory process and disease activity in various diseases have been reported. Studies have demonstrated that the monocyte/lymphocyte ratio, the platelet/lymphocyte ratio, and the neutrophil/lymphocyte ratio exhibit associations with several diseases.^[37,43]^ Studies demonstrated that neutrophil/lymphocyte ratio, platelet/lymphocyte ratio, and neutrophil/lymphocyte ratio were significantly correlated with the prognosis of patients with severe COVID-19^[17,24,44]^ In the present study, while the neutrophil/lymphocyte ratio and platelet/lymphocyte ratio were increased, the lymphocyte/monocyte ratio was decreased in our study.

The limitations of this study include its small sample size. Additionally, data were obtained from a single clinical research center and not from more than 1 clinical research center. Finding unvaccinated patients and healthy controls is challenging because the immune response generated by the vaccine may affect neopterin levels. Although the study was designed prospectively, early steroid treatment before the patient was transferred to the ICU, according to Turkish COVID-19 guidelines, could affect neopterin levels.

## 5. Conclusion

The findings from the present study showed that in patients with severe COVID-19, neopterin, CRP, albumin, the CRP-to-albumin ratio, the neutrophil/lymphocyte ratio, the platelet/lymphocyte ratio, and the lymphocyte/monocyte ratio could be potential parameters for indicating inflammation in patients with severe COVID-19. The other important finding in our study is that the neopterin levels significantly differed in patients with severe COVID-19 from healthy volunteers. Correlation analyses among these inflammatory biomarkers showed a correlation between neopterin levels and CRP and albumin levels in COVID-19 patients. These findings indicate that neopterin is equally crucial as conventional biomarkers in identifying inflammatory diseases, including COVID-19.

## Author contributions

**Conceptualization:** Sinem Gurcu, Ali Uncu.

**Data curation:** Sinem Gurcu, Zeynep Irmak Kaya.

**Methodology:** Sinem Gurcu.

**Resources:** Sinem Gurcu, Zeynep Irmak Kaya.

**Writing – original draft:** Sinem Gurcu, Göknur Yorulmaz.

**Writing – review & editing:** Sinem Gurcu, Göknur Yorulmaz, Sinem Ilgin.

**Formal analysis:** Ali Uncu.

**Supervision:** Göknur Yorulmaz, Sinem Ilgin.
